# Current challenges in thermodynamic aspects of rubber foam

**DOI:** 10.1038/s41598-021-85638-z

**Published:** 2021-03-17

**Authors:** Supitta Suethao, Worachai Ponloa, Saree Phongphanphanee, Jirasak Wong-Ekkabut, Wirasak Smitthipong

**Affiliations:** 1 Specialized Center of Rubber and Polymer Materials in Agriculture and Industry (RPM), Department of Materials Science, Faculty of Science, Kasetsart University, Bangkok, 10900 Thailand; 2 Department of Physics, Faculty of Science, Kasetsart University, Bangkok, 10900 Thailand; 3 Office of Research Integration on Target-Based Natural Rubber, National Research Council of Thailand (NRCT), Bangkok, 10900 Thailand; 4 Office of Natural Rubber Research Program, Thailand Science Research and Innovation (TSRI), Bangkok, 10900 Thailand

**Keywords:** Biophysics, Materials science, Mathematics and computing

## Abstract

Natural rubber (NR) foam can be prepared by the Dunlop method using concentrated natural latex with chemical agents. Most previous studies have focused on the thermodynamic parameters of solid rubber in extension. The main objective of this study is to investigate the effect of the NR matrix concentration on the static and dynamic properties of NR foams, especially the new approach of considering the thermodynamic aspects of NR foam in compression. We found that the density and compression strength of NR foams increased with increasing NR matrix concentration. The mechanical properties of NR foam were in agreement with computational modelling. Moreover, thermodynamic aspects showed that the ratio of internal energy force to the compression force, F_u_/F, and the entropy, S, increased with increasing matrix concentration. The activation enthalpy, ∆H_a_, also increased with increasing matrix concentration in the NR foam, indicating the greater relaxation time of the backbone of the rubber molecules. New scientific concepts of thermodynamic parameters of the crosslinked NR foam in compression mode are proposed and discussed. Our results will improve both the knowledge and the development of rubber foams based on the structure–properties relationship, especially the new scientific concept of the thermodynamical parameters under compression.

## Introduction

Polymer foams are formed by adding gas bubbles to the polymer matrix, leading to porosity and polymer continuous phases. The porosity, otherwise known as the cells, can be classified from its structure as either open–cell or closed–cell. The open–cell foam drives the flexibility, whereas the closed–cell foam drives the rigidity. Therefore, product applications depend on the structural properties of the foam^1–4^.

Natural rubber (NR) foam is an interesting natural polymer foam which can be made into lightweight products and is suitable for comfort applications such as pillows and mattresses. Generally, rubber foams are porous, elastic and have a ventilated surface^5,6^. Rubber foam has good elasticity^7^, and its mechanical properties can be tuned with the choice of the type of latex, foam structure, and filler loading, for example^7–10^.

The most important characteristic of a rubber material is that it consists of long flexible molecules^11^. Rubber molecules have a backbone of many covalent bonds which can rotate rapidly because of thermal agitation. Such long molecules convert their form easily at specific temperatures due to Brownian motion^12,13^. When no force is applied, they make random conformations but may adopt specific conformations if an external force is loaded. Changes in rubber elasticity are associated with changes in the configurational entropy and the system's internal energy during the deformation process^7^. For any elastic mechanism in extension mode, the stretching force is proportional to the temperature at a given state of strain. The thermodynamic study of the elastic deformation in rubber has shown that the stress depends significantly on the temperature in the extended state. It is possible to derive the separate contributions of the internal energy and entropy to the deformation process. The early experiments of Meyer and Ferri^14^ showed this statement to be substantially correct over a wide range of temperatures, provided that the extension was sufficiently large. The elastic behaviour seems to be irregular at lower strains because the force increases more slowly than would be expected from the theory. The deviations from the theoretical form of the force–extension curve for mechanical extension have been extensively studied by previous workers^15–17^; however, Mooney and Rivlin proposed a semi–empirical formula consistent with the experiment, referred to as the Mooney–Rivlin equation^18,19^.

When non–Gaussian theory^14,16^ has been applied to the experimental data at high strain process, the possible effects of strain–induced crystallisation on the mechanical properties of solid rubber have not been taken into account. The non–Gaussian theory of the strain state is significant within the crystallisation region, and it has been suggested that the rise of the force–deformation curve should be attributed primarily to this factor^20^. The crystallisation that occurs would certainly be expected to produce some stiffening of the rubber, but it is not easy to predict the magnitude of the effect. However, it has been noted by Wang and Guth^21^ that the characteristic form of the force–extension curve for natural rubber is only slightly affected by increasing the temperature to 100 °C, though this will substantially reduce the crystallinity. Most existing studies investigated the solid rubber in extension mode; fewer studies have focused on the thermodynamics of polymer foams, especially rubber foam^7,22^. In the present work, a new scientific concept of the thermodynamic parameters of the natural rubber (NR) foam is investigated. The mechanical properties are modelled. In particular, we consider some features of the compression of crosslinked rubber foams which are still not fully understood.

## Materials and methods

### Materials

The concentrated natural latex (60% dry rubber content and 1.7% non–rubber content), from *Hevea brasiliensis* in the southern part of Thailand, was supplied from Num Rubber & Latex Co., Ltd., Trang, Thailand. The chemical agents consist of 10% potassium oleate solution, 50% sulphur dispersion, 50% zinc diethyldithiocarbamate (ZDEC) dispersion, 50% zinc–2–mercaptobenzothiazole (ZMBT) dispersion, 50% Wingstay L dispersion, 50% zinc oxide (ZnO) dispersion, 33% diphenylguanidine (DPG) dispersion, and 12.5% sodium silicofluoride (SSF) dispersion. All the chemical agents were supplied from Thanodom Technology Co., Ltd., Thailand. The chemical agents used for the rubber foam preparation are summarised in Table 1.

**Table 1:** Chemical agents used for the preparation of various foam samples.

Chemical agents	Control + 10% NR or control added 10% NR (g)	Control (g)	Control − 10% NR or control reduced 10% NR (g)
60% concentrated natural latex	183.33	166.67	150.00
10% potassium oleate solution	16.50	16.50	16.50
50% sulphur dispersion	4.00	4.00	4.00
50% ZDEC dispersion	2.00	2.00	2.00
50% ZMBT dispersion	2.00	2.00	2.00
50% Wingstay L dispersion	2.00	2.00	2.00
50% ZnO dispersion	10.00	10.00	10.00
33% DPG dispersion	2.00	2.00	2.00
12.5% SSF dispersion	8.00	8.00	8.00

### Rubber foam preparation

The rubber foams were prepared in the following way: first, concentrated natural latex was stirred 80 rpm in a blender for 1 min to remove the ammonia. Second, the potassium oleate solution was added as the stirring speed was increased to 160 rpm for 10 min. Then, dispersions of sulphur, ZDEC, ZMBT and Wingstay L were added to the rubber compound with the stirring speed decreased to 80 rpm for 1 min. Next, ZnO and DPG dispersions were added into the rubber compound at the same mixing speed for 1 min. After that, the SSF dispersion was added into the rubber compound and mixing continued until the rubber foam had nearly reached the gel point. Finally, the rubber foam was transferred to a mould and allowed to set for 45 min. The vulcanisation of the rubber foam was performed by a hot air oven at 90 °C for 2 h. The rubber foam was then removed from the mould, washed, and dried in the hot air oven at 70 °C for 4 h.

### Rubber foam characterisation

The density (kg/m^3^) of the foam was evaluated by the relationship between the weight (kg) and volume (m^3^) of the foam as described elsewhere^9^.

The chemical functional groups present in the foam sample were measured by Attenuated Total Reflection–Fourier Transform Infrared (ATR–FTIR) spectroscopy with a Ge crystal probe (VERTEX 70, Bruker, Billerica, MA, USA).

The morphology of foam samples was examined by a scanning electron microscope (SEM, FEI, Quanta 450, Eindhoven, Netherlands). The foam sample was coated with gold, and three replicants of each foam formula were tested. ImageJ software^23^ was used to evaluate the average pore size and porosity of the foam samples. The cell density (d_cell_) of the foam sample was calculated as in a previous study^7^ for comparison with the solid phase density of natural rubber (NR 0.93 g/cm^3^).

The compression stress of a foam sample as a function of strain was determined by a texture analyser (TA.XT Plus, Stable Micro Systems, Godalming, Surrey, UK) with a platen probe of 100 mm diameter at 0.1 mm/sec and room temperature.

The computational modelling of the mechanical properties of the foam sample used the hyperfoam–polynomial strain energy function from 1st to 6th order^24^. The finite element method (FEM) and curve–fitting analysis of foam sample data were performed using ABAQUS^25^ under uniaxial compression.

The relationship between the stress, σ, and the compression limit, λ, of each foam sample was plotted on the Mooney–Rivlin Eq. ^26^ where C_1_ and C_2_ were the constant values:

1 $$ \frac{\upsigma }{{\left( {\uplambda - \frac{1}{{\uplambda ^{2} }}} \right)}} = 2{\text{C}}_{1} + 2{\text{C}}_{2} \frac{1}{\uplambda } $$

The crosslinking density of the foam samples was evaluated by the swelling method according to the Flory–Rehner equation^27–31^. We also used the Flory–Huggins equation to calculate the change in Gibbs free energy, ∆G, and entropy, ∆S, as follows^32,33^:

2 $$ \Delta {\text{G}} = {\text{RT}}\left[ {{\text{ln}}\left( {1 - {\text{V}}_{{\text{r}}} } \right) + {\text{V}}_{{\text{r}}} + {\text{V}}_{{\text{r}}}^{2} } \right] $$

3 $$ \Delta {\text{S}} = - \frac{{\Delta {\text{G}}}}{{\text{T}}} $$

where V_r_ is the volume fraction of foam sample in the rubber network, χ is the parameter between the foam sample and the solvent interaction (defined as 0.43 + 0.05 V_r_)^28^, R is the ideal gas constant (8.3145 J/mol·K), and T is the test temperature (298.15 K).

The thermodynamic parameters of the foam sample compression were measured by the texture analyser (TA.XT Plus, Stable Micro Systems, Godalming, Surrey, UK). The foam sample was also incubated at different temperatures (298.15, 308.15, 318.15, 328.15, and 338.15 K) during the compression process from 10 strain to 70% strain. Next, the relationship between force and temperature was plotted to obtain the ratio of internal energy to the compression force, F_u_/F.

The activation enthalpy of the transition process, ∆H_a_, of the foam sample was evaluated by dynamic mechanical analysis (DMA1, Mettler Toledo, Columbus, OH, USA) from − 193.15 to 353.15 K. From this, the ∆H_a_ value of the foam sample can be calculated as described elsewhere^33^.

## Results and discussion

Here we investigate the underlying thermodynamic relations of these rubber foam phenomena in more detail and explore how they may be applied experimentally to obtain quantitative information about the deformation process mechanism.

### Physical and morphological properties

**Figure 1:**
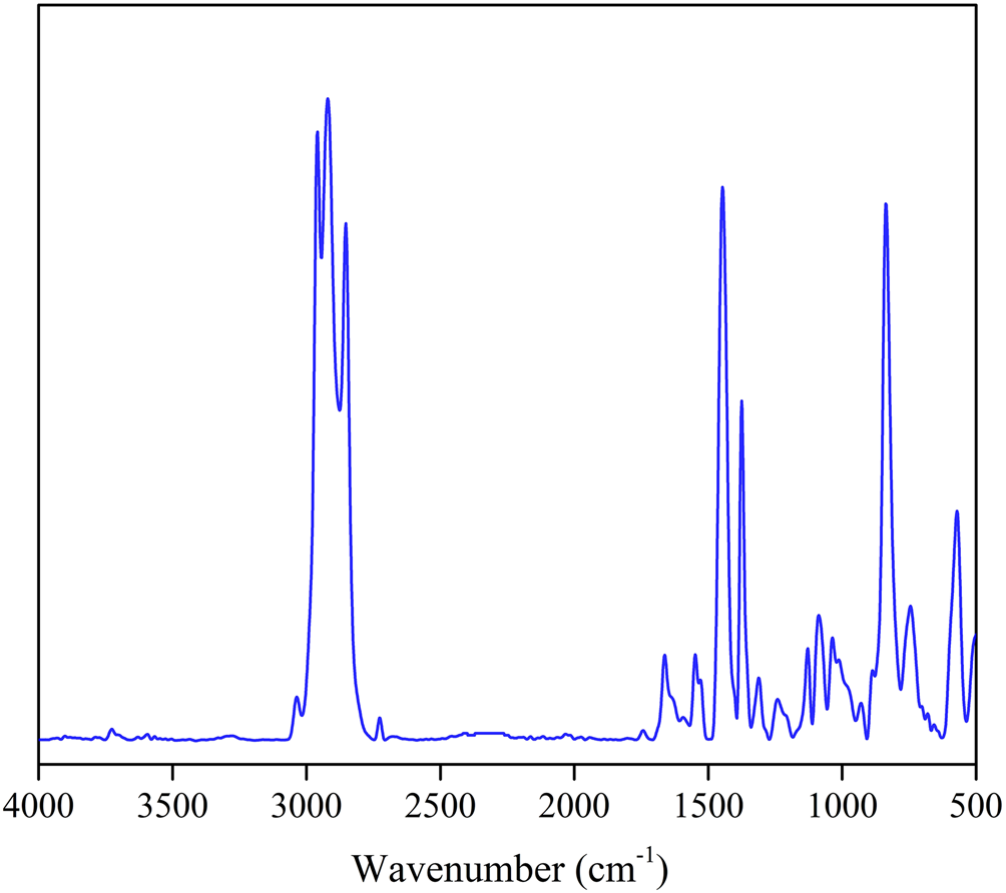
ATR–FTIR spectrum of the control sample: 3015–2970 cm^−1^ attribute to C–H stretching of CH_3_ and CH_2_, 1672 cm^−1^ attributes to C=C stretching, 935–1171 cm^−1^ attribute to C–S stretching, and 840 cm^−1^ attributes to C=C wagging^9^.

First, we discuss the chemical function of the rubber foams. Figure 1 presents an ATR–FTIR spectrum of the control sample^7,9^ from 500 to 4000 cm^−1^, this result confirmed the chemical functional group of crosslinked NR foam. However, there are no significant differences between the spectra of the foam samples, even at different rubber concentrations. To investigate the density of the rubber foams (Table 2), rubber foam samples of each type with the same volume (4.86 × 10^–5^ m^3^) were prepared. Each rubber foam sample was weighed in kilograms. The results showed that the amount of matrix has a significant effect on the density of the foam sample. The density of the foam sample decreased by around 10% when the matrix was reduced by 10%, while the addition of 10% matrix content increased the density of the foam sample of around 10%.

**Table 2:** Foam density, average pore size, porosity, and cell density of various foam samples.

Sample	Foam density (± 3 kg/m^3^)	Average pore size (± 150 µm)	Porosity (± 1.00%)	Cell density (± 500 cm^-3^)
Control + 10% NR	123	352	45.97	38,054
Control	110	522	48.73	11,844
Control − 10% NR	99	555	55.37	9,966

**Figure 2:**
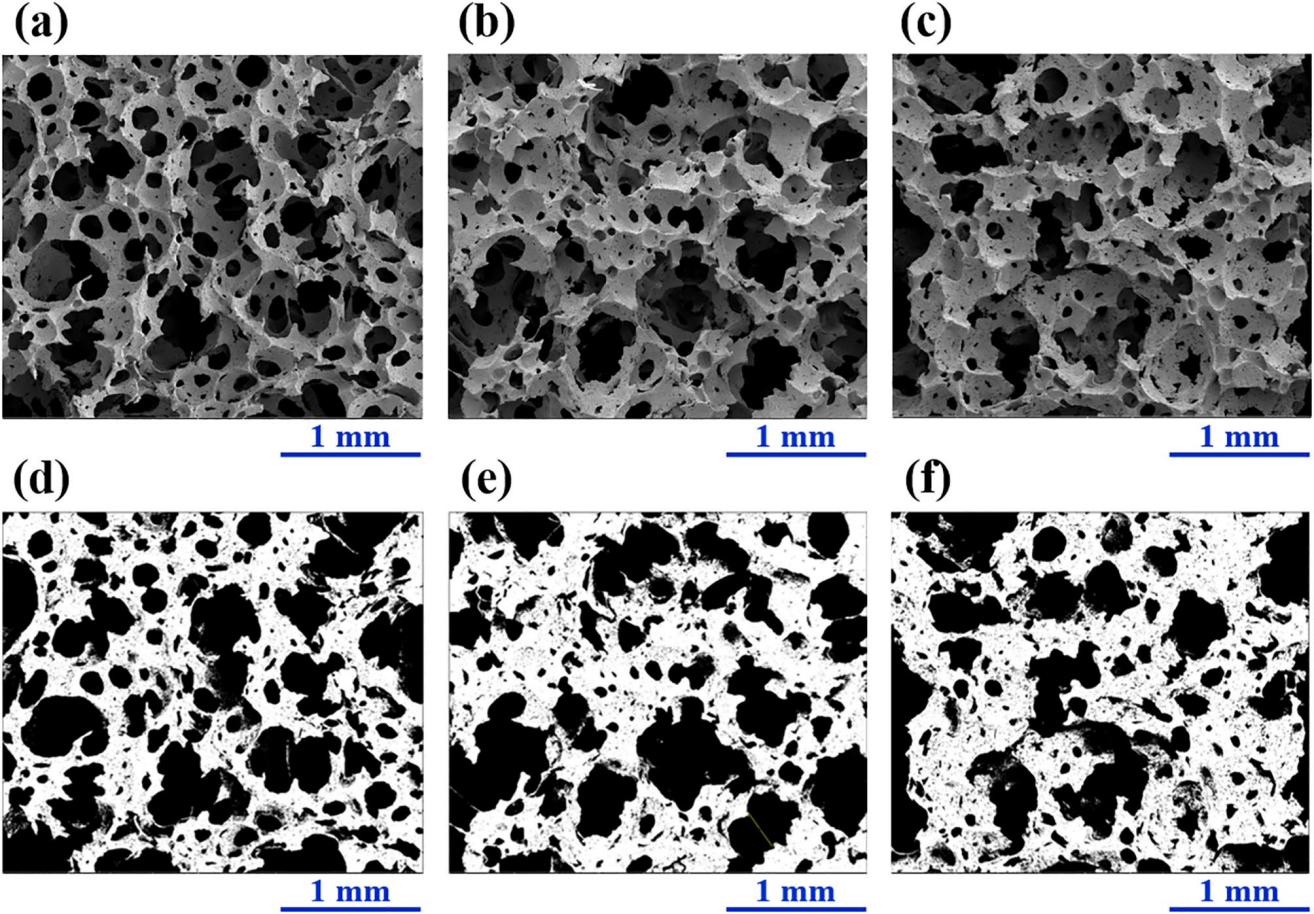
SEM images of the various foam samples at 50× magnification: (**a**) control − 10% NR, (**b**) control, and (**c**) control + 10% NR. Images of various foam samples from ImageJ analysis: (**d**) control − 10% NR, (**e**) control, and (**f**) control + 10% NR. The white areas are related to the interconnected foam, whereas the black areas relate to the pore or cell.

In terms of the morphological properties, the images obtained from the SEM are presented in Fig. 2. We found that all the foam samples exhibited the open–cell structure with heterogeneous sizes of bubble (heterogeneous pore size), a known effect of the Dunlop process^34,35^. However, the amount of NR matrix present affects the morphological properties of the foam samples. The foam of samples with a higher NR concentration is more interconnected (Fig. 2a–c), which agrees with the density measurements. The ImageJ software^23^ was used to evaluate the morphological parameters of the foam samples (Fig. 2d–f and Table 2). In Fig. 2d–f, the white areas are related to the interconnected foam, while the black areas relate to the pore or cell. The average pore size and porosity calculated by ImageJ analysis decrease with the increasing amount of NR matrix (Table 2). This trend is in good agreement with other works investigating the effects of filler concentration and the type of NR matrix on the properties of foams^7,9^. The cell density, calculated in a previous study^36^, is in good agreement with the SEM images and ImageJ analysis results. The cell density of the foam samples increases with increasing NR matrix concentration. However, the present values of cell density are higher than those of our previous work^7^, because foam samples used in our current study have higher densities than in the previous study.

### Mechanical properties

**Figure 3:**
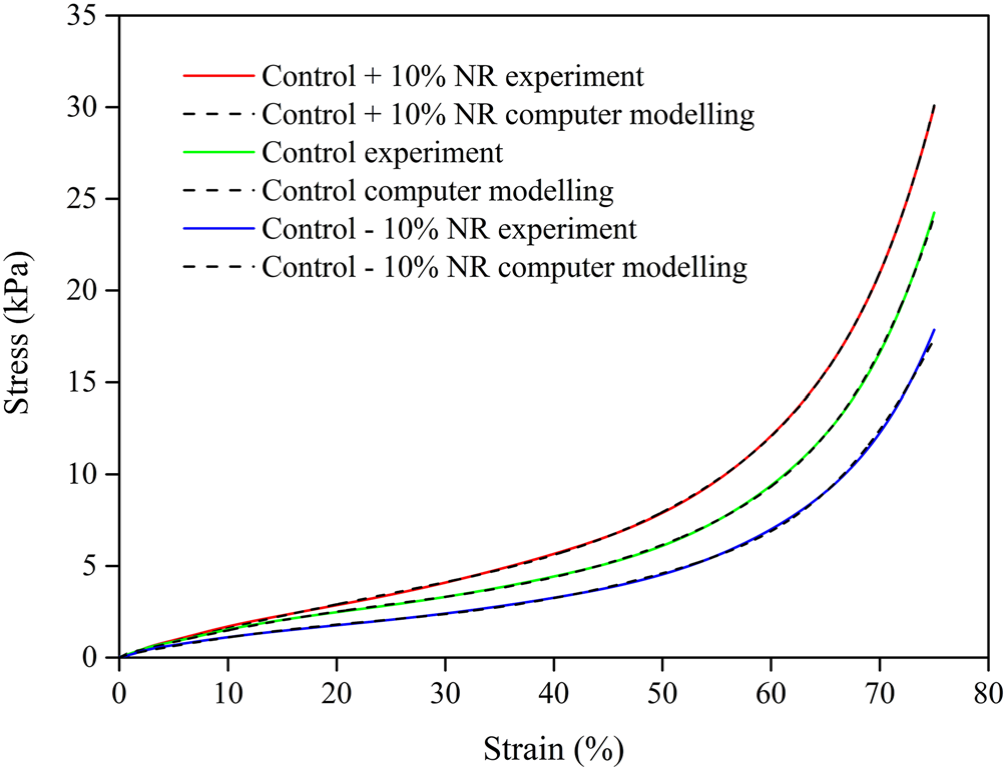
Compression stress–strain curves of foam samples from the experimental data and the computation of the 6th order reduced polynomial model.

Figure 3 shows the stress–strain curves from experiments and hyperfoam material modelling of rubber foam samples. For the reduced polynomial model used in this study, the 6th order is in good agreement with the experimental result. Table 3 presents the parameters from the fit to the 6th order, and the computer modelling of the foam samples in compression is in good agreement with the experimental results (Fig. 3). Concerning the compression strength, which is the maximum compression stress at 75% from the rubber foam surface, we found that the compression strength increases with increasing NR matrix content. This result is in agreement with the density of the foam samples. Generally, NR exhibits good mechanical properties because it has a high molecular weight^8,37^. Interestingly, the mechanical property, compression strength, is more sensitive to the matrix content than the density. The compression strength of rubber foam is decreased around 37% by a 10% reduction in matrix content while 10% addition to the matrix content results in an increase of compression strength of around 23%. In general, the compressive axial tests of foams show three main distinct regions^38^. The deformation process starts with an initial linear elastic response on cell edges or cell walls. Deformations are increasing and leading to the cell starts to collapse while stresses remain roughly unchanged, known as the plateau region. This effect results in the ability to absorb impact and vibrating loading. This collapse progresses until opposing walls meet and touch. After the opposing walls touch, the deformation stops with increasing stresses (densification or locking) i.e. cellular solids exhibit deformation until the densification is reached. Alzoubi et al.^39^ showed that the natural latex is highly elastic and low viscous characteristics than any other foam samples such as Polyurethane. Therefore, the latex material has no clear distinct three regions as the case for other samples. However, at high strains, the cell walls of NR foam are completely collapsed: higher compression strength is represented by a higher concentration of the NR matrix.

**Table 3:** Modelling parameters from the 1st to 6th order of the reduced polynomial for the foam samples.

Sample	C_10_ (10^–2^)	C_20_ (10^–5^)	C_30_ (10^–9^)	C_40_ (10^–12^)	C_50_ (10^–16^)	C_60_ (10^–20^)	$\upmu _{0}$(10^–1^)
Control + 10% NR	5.47	− 0.84	2.62	− 0.03	− 0.44	0.59	1.10
Control	7.33	− 1.93	8.47	− 1.78	2.04	− 0.78	1.47
Control − 10% NR	5.53	− 1.86	9.43	− 2.32	3.00	− 1.42	1.11

**Figure 4:**
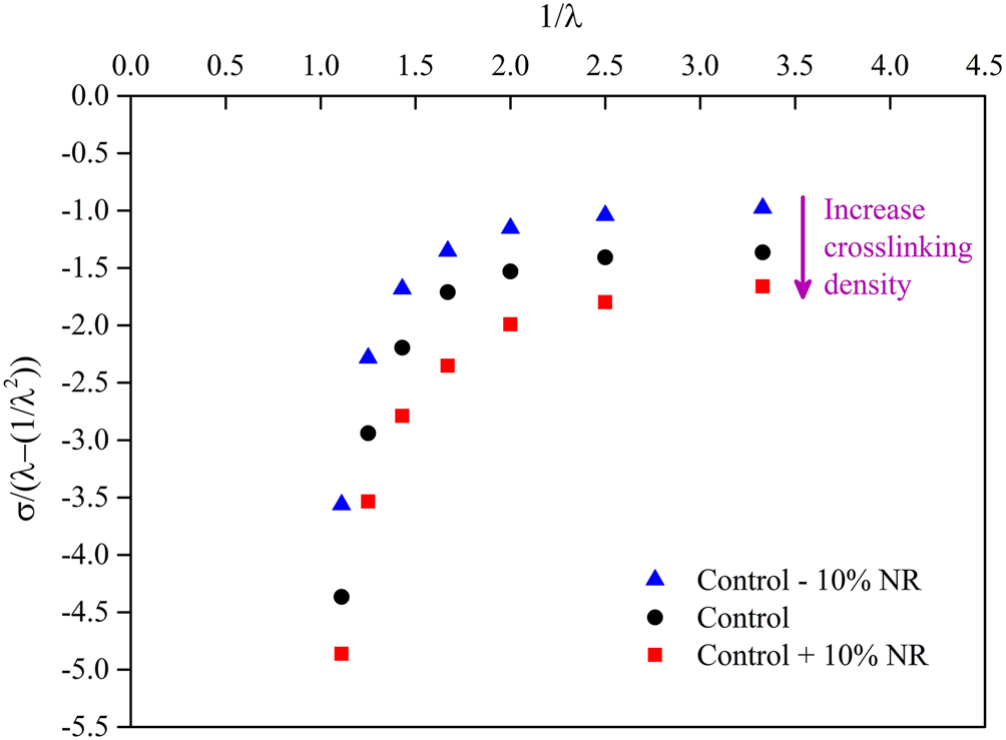
The relationship of stress, σ, and compression limit, λ, of foam samples based on the Mooney–Rivlin Eq. ^26^. The relative scatter on the results is estimated equal to about 5%.

The mechanical properties of rubber foam can also be investigated in terms of the stress, σ, and the compression limit, λ, based on the Mooney–Rivlin equation^26^ (Fig. 4). We found that the slope of the straight line above 1.5 at x–axis for all samples is almost identical. The y–intercept for all samples is related to the crosslinking density of the foam samples; higher crosslinking density is represented by a higher absolute value of the y–intercept. The resulting pattern of curves is in good agreement with the Mooney–Rivlin experiment^25^, although our curves were in the opposite direction compared to the Mooney–Rivlin result. However, the Mooney–Rivlin result was obtained in extension mode of solid rubber while our study was in compression mode of rubber foam.

### Thermodynamic aspects

Based on the crosslinking density of the foam samples, a foam with higher NR matrix concentration possesses a higher volume fraction of rubber, V_r_, and greater crosslinking density. We can calculate the change in the Gibbs free energy, ∆G, and entropy, ∆S, from the swelling test based on the Flory–Huggins equation^32,33^; results are given in Table 4. A negative ∆G was found for all the foam samples, and the ∆G decreases with increasing NR matrix concentration. Moreover, ∆S increases with increasing matrix concentration, which indicates favourable thermodynamics. This is because of the NR matrix concentration on the foam sample: rubber with good mechanical properties (high compression strength and modulus) and high relaxation stress result in a more thermodynamically favourable system^7,16^.

**Table 4:** Thermodynamic parameters determined from the crosslinking density of various foam samples based on the Flory–Huggins equation^32,33^.

Sample	Volume fraction of rubber, V_r_ (± 0.001% a.u.)	ΔG (J/mol)	ΔS (J/mol K)
Control + 10% NR	0.2605	− 36.7143	0.1231
Control	0.2595	− 36.3674	0.1220
Control − 10% NR	0.2575	− 35.6386	0.1195

**Figure 5:**
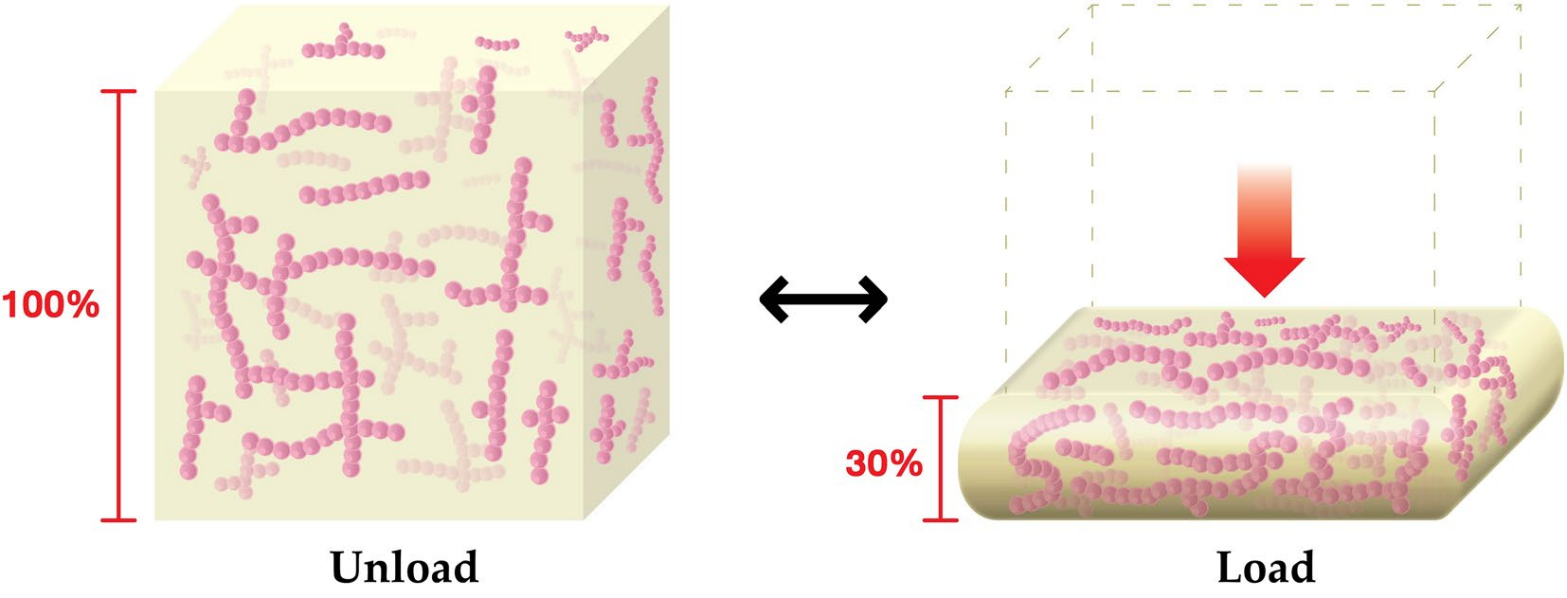
Unloaded (left) and loaded at z–direction (right) models of rubber foam samples. Unloaded rubber molecules (pink lines) have freedom to move, whereas loaded rubber molecules cannot move and rubber foam is expanded at x– and y–directions due to the compression. The model was drawn using Adobe illustrator software vision 24.2.3^40^.

It is extremely interesting to investigate the thermodynamic aspects of rubber foam related to the mechanical properties in more detail. We focused on the compression of the foam samples. The compression force came exclusively from an entropic mechanism, for example, from the tendency of the rubber molecule to transform to random conformations. The compression force was then directly proportional to the absolute temperature^14–16^. Figure 5 illustrates the model of the unloaded foam sample, which corresponds to a high degree of freedom for the rubber molecules, and the model of the loaded foam sample from compression, which corresponds to a lower degree of freedom for the rubber molecules. When the compression force is unloaded, the foam sample returns to its original shape, which is more favourable in terms of the entropy, S, of the entangled molecules.

Consider the consequence of an external force causing compression on a rubber foam. The first law of thermodynamics can be written^41^:

4 $$ {\text{dU}} = {\text{dQ}} + {\text{dW}} $$

where dU is the change in the rubber foam’s internal energy resulting from the absorption of heat, dQ, and the distribution of work, dW, on it by the external force. If we assume that the compression process is reversible based on the porous structure of rubber foam, the heat flow can be expressed as^41^:

5 $$ {\text{dQ}} = {\text{TdS}} $$

where T is the temperature and dS is the change in entropy, and thus:

6 $$ {\text{dU}} = {\text{TdS}} + {\text{dW}} $$

This equation concerns the reversible compression of a foam sample. The work is done by applying a force, F, to the foam, resulting in the change of length, dL, from its original length. From the perspective of porous structure of rubber foam, when a foam is compressed at the z–direction, it can be expanded at the x– and y–directions. So, the uniaxial work done on the foam based on the assumption of constant volume is thus:

7 $$ {\text{dW}} = {\text{FdL}} $$

Then, we combine (4), (5), (6), and (7) and produce:

8 $$ {\text{dU}} = {\text{TdS}} + {\text{FdL}} $$

We can take the partial differential of (8) with respect to L as follows^14,16^:

$$ \frac{{{\text{dU}}}}{{{\text{dL}}}} = {\text{T}}\frac{{{\text{dS}}}}{{{\text{dL}}}} + {\text{F}} $$

9 $$ {\text{F}} = \frac{{{\text{dU}}}}{{{\text{dL}}}} - {\text{T}}\frac{{{\text{dS}}}}{{{\text{dL}}}} = {\text{F}}_{{\text{u}}} + {\text{F}}_{{\text{s}}} $$

where F_u_ = dU/dL and F_s_ = − T(dS/dL). These two thermodynamic parameters relate to the internal energy and entropy changes on compression of the rubber foam sample.

**Figure 6:**
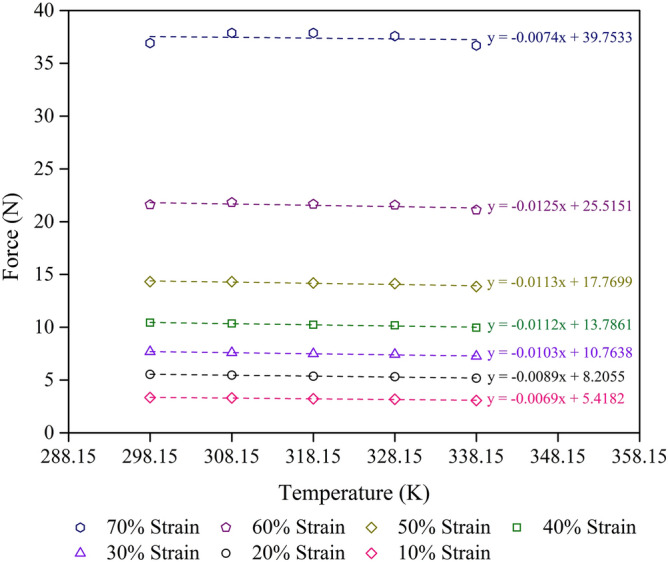
Force–temperature curves at a given strain for the control + 10% NR sample with R^2^ = 0.9 minimum at each strain. The scatter on the results is on the order of the size of the figures.

**Figure 7:**
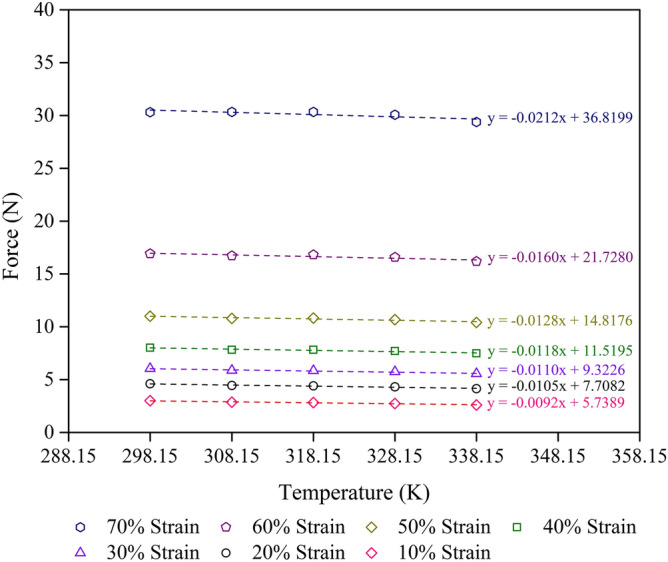
Force–temperature curves at a given strain for the control sample with R^2^ = 0.9 minimum at each strain. The scatter on the results is on the order of the size of the figures.

**Figure 8:**
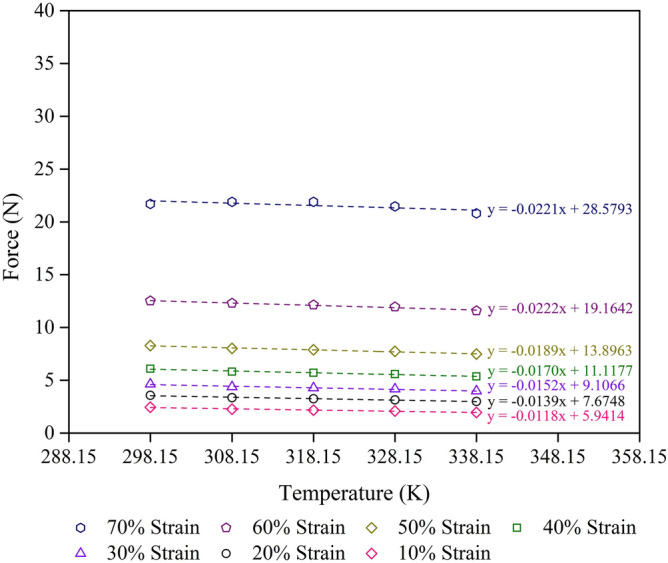
Force–temperature curves at given strain for the control − 10% NR sample with R^2^ = 0.9 minimum at each strain. The scatter on the results is on the order of the size of the figures.

Equation (9) is of basic significance in rubber elasticity since it provides a direct measurement of the changes of the internal energy and the entropy during a deforming. Its application is illustrated by Figs. 6, 7 and 8, in which the linear curve represents the variation with temperature of the force at a constant compressive strain. From (9), the slope of this curve gives the entropy change per unit compression, dS/dL, for isothermal compression at the temperature T. Correspondingly, the y–intercept at T = 0 is dU/dL, the change of internal energy per unit compression.

Thus, the internal energy and entropy contributions to the force at any given compression strain can be obtained by the experimental force–temperature curve of the foam samples with different compression strains and temperatures (Figs. 6, 7, 8). The internal energy and entropy terms are independent of the temperature when the force–temperature curve is linear. However, at high compression strains, the effect of crystallisation could become significant. Strain–induced crystallisation of solid uncrosslinked rubber has previously been reported^42,43^, while stress–induced crystallisation of crosslinked rubber foam has been reported^7^. Figures 6,7 and 8 show the increase of compression force with increasing compression strain (from 10 to 70% strain) in all types of foam sample studied. At a certain compression strain, the compression force seems to be stable, indicating the high–density foam samples which are in good agreement with a previous study on high–density rubber foams with silica filler^7^. Furthermore, the slope decreases slightly at higher strains, indicating the decrease degree of freedom of the rubber molecules.

Concerning the elasticity of the foam sample in compression mode (Figs. 6, 7, 8), samples under high length or low compression strain, L, exhibits high entropy, S, values. Thus, the entropy of a foam sample is proportional to the length at a constant temperature, as shown in Eq. (10).

10 $$ {\text{S}} \propto {\text{L}} $$

**Figure 9:**
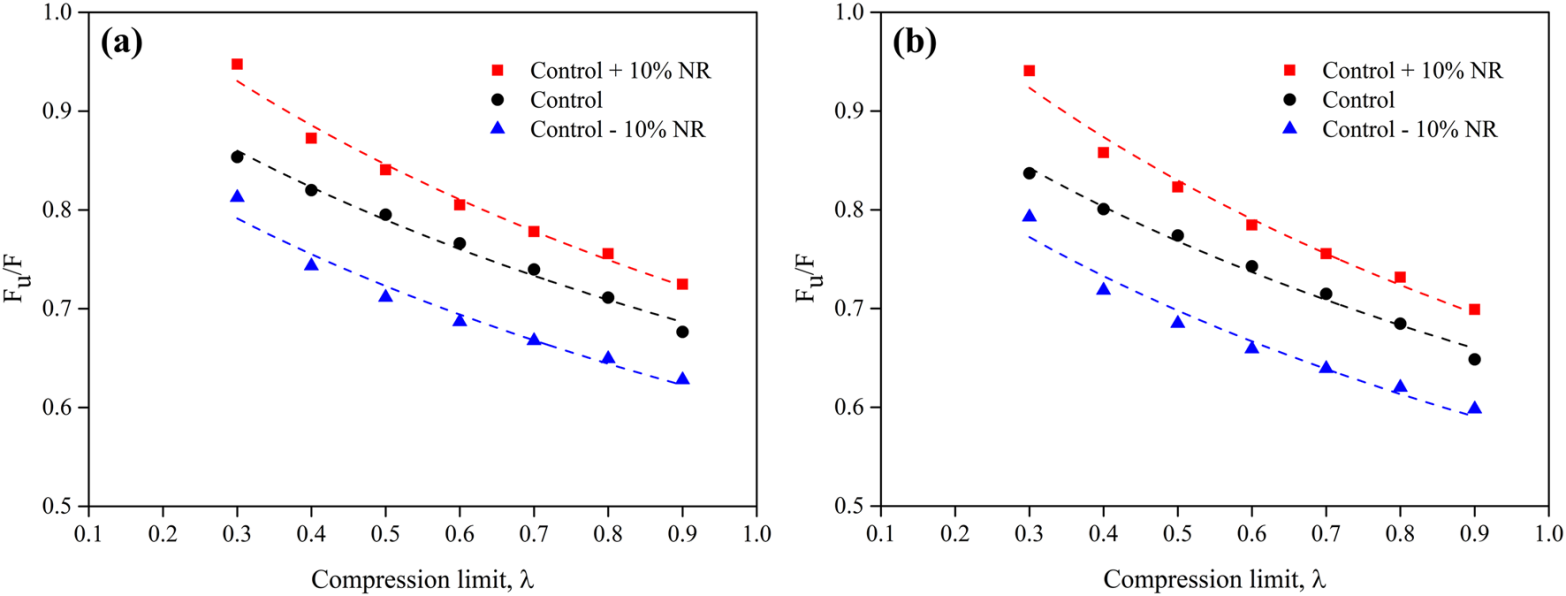
Internal energy contribution vs the compression force, F_u_/F, of various foam samples: (**a**) 298.15 K, and (**b**) 338.15 K. The scatter on the results is on the order of the size of the figures.

Table 5 shows the values of the F_u_/F ratio of various foam samples at different compression strains and two temperatures (298.15 and 338.15 K). The values of F_u_ and F grow as the compression strain increases for all types of foam sample. Moreover, the F_u_/F value also increases with increasing compression strain, indicating the stability of the entropy during the deformation process. This result agrees with previous work on rubber foam with high silica loading, which showed high density and better mechanical properties^7^. While the NR matrix concentration affects the mechanical properties of the foam samples, the ratio of F_u_/F relates to the thermodynamic aspects of the mechanical properties of the foam samples. This result is in good agreement with the calculations of ∆G and ∆S. Moreover, the ratio of F_u_/F increases as the temperature decreases from 338.15 to 298.15 K. This can be explained by the effect of the flow property of natural rubber at higher temperatures^44,45^. The F_u_/F values obtained from this study (0.7–0.9) are higher than those from previous studies: 0.1–0.2 values for uncrosslinked rubber in extension^16^ and 0.6–0.8 values for lower density crosslinked foam samples in compression^7^. The difference in F_u_/F values could be due to the differences in the rubber structure and the test method used. Figure 9 shows the relationship between F_u_/F and the compression limit, λ, of various foam samples at 298.15 K and 338.15 K. We found that the slope is similar to that from a previous study of rubber foams with a large amount of silica loading^7^. The slope direction of the foam samples is not significantly different between the two temperatures. However, the NR matrix concentration in the foam sample affects the level of the F_u_/F graphs because of the mechanical properties of the foam sample.

**Table 5:** Compression strain, F_u_, F, and F_u_/F values of foam samples at 298.15 K and 338.15 K.

Sample	Compression strain (%)	F_u_ (± 5% N)	298.15 K		338.15 K	
			F (± 5% N)	F_u_/F	F (± 5% N)	F_u_/F
Control + 10% NR	10	5.42	7.48	0.7248	7.75	0.6990
	20	8.21	10.86	0.7556	11.22	0.7317
	30	10.76	13.83	0.7780	14.25	0.7555
	40	13.79	17.13	0.8050	17.57	0.7845
	50	17.77	21.14	0.8406	21.59	0.8230
	60	25.52	29.24	0.8726	29.74	0.8579
	70	39.75	41.96	0.9474	42.26	0.9408
Control	10	5.74	8.48	0.6766	8.85	0.6485
	20	7.71	10.84	0.7112	11.26	0.6846
	30	9.32	12.60	0.7398	13.04	0.7148
	40	11.52	15.04	0.7660	15.51	0.7427
	50	14.82	18.63	0.7952	19.15	0.7739
	60	21.73	26.50	0.8200	27.14	0.8006
	70	36.82	43.14	0.8535	43.99	0.8370
Control − 10% NR	10	5.94	9.46	0.6281	9.93	0.5982
	20	7.67	11.82	0.6494	12.38	0.6202
	30	9.11	13.64	0.6677	14.25	0.6392
	40	11.12	16.19	0.6869	16.87	0.6592
	50	13.90	19.53	0.7115	20.29	0.6850
	60	19.16	25.78	0.7433	26.67	0.7185
	70	28.58	35.17	0.8126	36.05	0.7927

**Figure 10:**
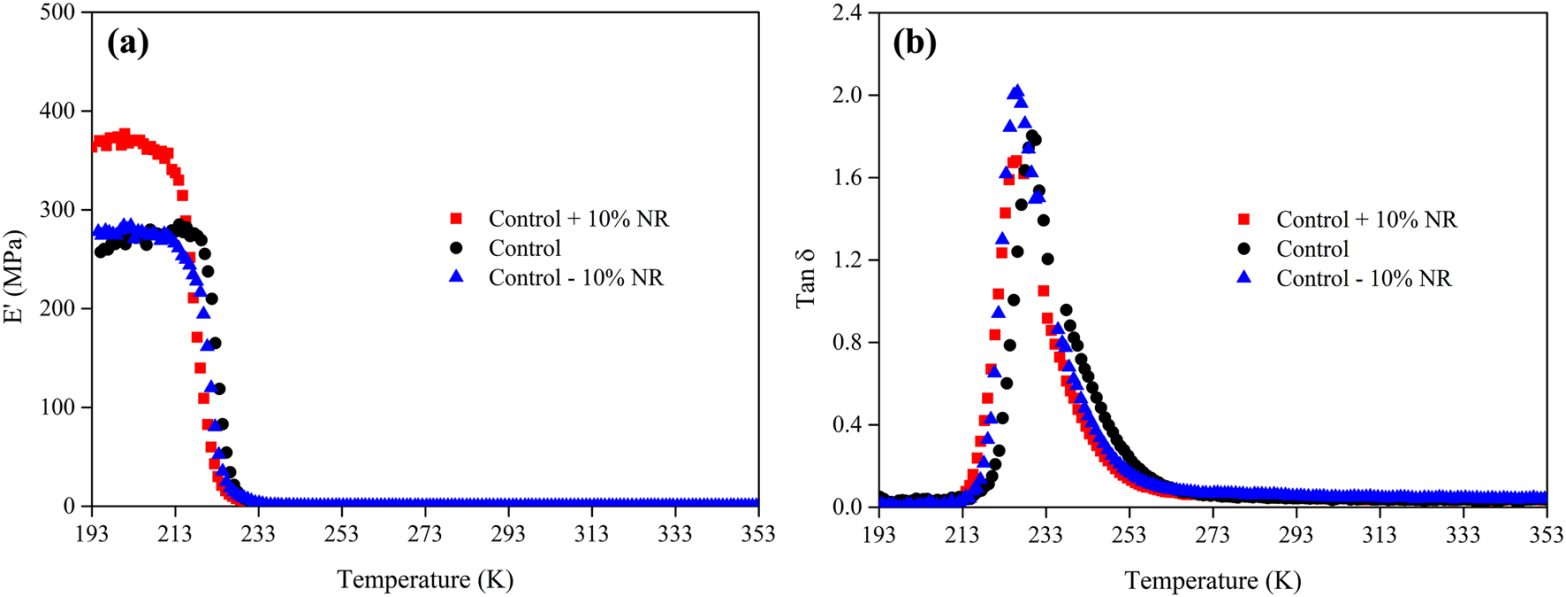
Dynamic mechanical analysis of various foam samples: (**a**) storage modulus, E**'**, as a function of temperature, and (**b**) tan δ as a function of temperature. The relative scatter on the results is estimated equal to about 5%.

The thermodynamic parameters of foam samples were also calculated from the perspective of dynamic mechanical analysis. The storage modulus, E′, and tan δ of the foam samples as a function of temperature are presented in Fig. 10. In general, the storage modulus relates to the dynamic mechanical properties whereas tan δ relates to the dissipation energy of a material^46,47^. Rubber chains are freezing at the glassy plateau below the glass transition temperature, the foam sample with high NR content (control + 10% NR) represents a high storage modulus at the glassy plateau, indicating the lower free volume for high density samples^46^. However, the storage modulus of other two samples is quite similar. The NR matrix concentration also affects the dissipation energy or hysteresis (maximum tan δ) of a foam sample. Samples with a high NR concentration possess more network structure, generating the low hysteresis. This result is in good agreement with the existing literature, and the hysteresis of the rubber foam could be due to either the molecular friction of short molecules or the reduced network structure^8,48^.

Table 6 presents the dynamic mechanical parameters extracted from Fig. 10, from which the activation enthalpy, ∆H_a_, of various foam samples can be calculated. This activation enthalpy focuses on the transition process between the glassy and rubber states of rubber molecules. This average value also corresponds to the relaxation of the backbone motion of rubber molecules^33^. Interestingly, the NR matrix concentration again affects the activation enthalpy of the foam sample, where a higher NR concentration represents a higher average activation enthalpy, ∆H_a_, due to the greater relaxation time of the rubber chains. These average activation enthalpy values are in good agreement with the previous literature^7,33^.

**Table 6:** Parameters obtained from the dynamic mechanical analysis of various foam samples.

Sample	E'_glassy_ at 203.15 K (± 5% MPa)	E'_rubber_ at 273.15 K (± 5% MPa)	T_g_ dynamic (K)	Tan ẟ max	t_A_ (area under tan ẟ peak)	∆H_a_ (kJ K/mol)
Control + 10% NR	337.28	0.37	232.07	1.68	33.44	143.36
Control	293.53	0.31	229.98	1.80	34.50	137.30
Control − 10% NR	324.73	0.49	226.65	2.02	33.22	131.28

## Conclusions

In this study, we applied the Dunlop process to prepare rubber foam samples with different NR matrix concentrations. Then we investigated the thermodynamic relations of the system in more detail and how they may be applied experimentally to obtain quantitative information during the compression process. We found that the NR matrix content has a significant effect on the density and compression strength of the foam sample; nevertheless, the compression strength is more sensitive to the matrix concentration than the density. The foam samples have an open–cell structure with heterogeneous cell sizes, samples with higher NR matrix concentration exhibit higher interconnectivity and cell density.

The computational modelling using the hyperfoam model of the 6th order is in good agreement with the experimental result of the foam samples in the stress–strain curve. Moreover, the mechanical properties of the foam samples with different matrix concentrations are in good agreement with that of the Mooney–Rivlin experiment. However, our work was in compression mode while Mooney–Rivlin’s was in extension mode.

Based on the crosslinking density of the foam sample, the higher NR matrix concentration results in a higher volume fraction of rubber, V_r_, and crosslinking density. When the Flory–Huggins equation is applied, the change in Gibbs free energy, ∆G, decreases, and the change in entropy, ∆S, increases with the increasing NR matrix concentration, which is thermodynamically favourable. The force and temperature relationship corresponded to the internal energy and entropy, which were experimentally determined by compression of foam samples. Interestingly, the ratio of the internal energy force to the compression force, F_u_/F, increases with the NR matrix concentration, and the F_u_/F ratio is in good agreement with the literature reviews. Thus, the change of foam length, ΔL, or compression strain, is directly influenced by the entropy change.

Dynamic mechanical analysis was applied to evaluate the activation enthalpy of the transition process, ∆H_a_, of the foam samples. The results showed that a higher NR matrix concentration has the effect of increasing the average activation enthalpy related to the relaxation time of rubber molecules. Therefore, the NR matrix concentration affects the static and dynamic parameters resulting from the relationship between the rubber foam structure and the material properties. New approaches in the thermodynamic aspects of foam samples related to the matrix concentration effect were investigated and proposed.

## Data Availability

Correspondence and requests for materials should be addressed to W.S.
